# Practical Physical and Behavioral Measures to Assess the Socialization Spectrum of Cats in a Shelter-Like Setting during a Three Day Period

**DOI:** 10.3390/ani3041162

**Published:** 2013-12-18

**Authors:** Margaret Slater, Laurie Garrison, Katherine Miller, Emily Weiss, Kathleen Makolinski, Natasha Drain, Alex Mirontshuk

**Affiliations:** 1 Shelter Research and Development, Community Outreach, American Society for the Prevention of Cruelty to Animals (ASPCA^®^), 50 Stone Ridge Drive, Florence, MA 01062, USA; 2 Shelter Research and Development, Community Outreach, American Society for the Prevention of Cruelty to Animals (ASPCA^®^), P.O. Box 408, Little Silver, NJ 07739, USA; E-Mail: laurie.garrison@aspca.org; 3 Shelter Research and Development, Community Outreach, American Society for the Prevention of Cruelty to Animals (ASPCA^®^), 520 Eighth Avenue, 7th Floor, New York, NY 10018, USA; E-Mail: katherine.miller@aspca.org; 4 Shelter Research and Development, Community Outreach, American Society for the Prevention of Cruelty to Animals (ASPCA^®^), 3201 SW Winding Way, Palm City, FL 34990, USA; E-Mail: emily.weiss@aspca.org; 5 Veterinary Outreach, Community Outreach, American Society for the Prevention of Cruelty to Animals (ASPCA^®^), P.O. Box 1144, Orchard Park, NY 14127, USA; E-Mail: kathleen.makolinski@aspca.org; 6 Shelter Research and Development, Community Outreach, American Society for the Prevention of Cruelty to Animals (ASPCA^®^), P.O. Box 4323, Arlington, VA 22204, USA; E-Mail: natasha.drain@aspca.org; 7 Shelter Research and Development, Community Outreach, American Society for the Prevention of Cruelty to Animals (ASPCA^®^), 1216 32nd Street, Oakland, CA 94608, USA; E-Mail: alexmm1345@gmail.com

**Keywords:** feral cat, animal shelter, behavior, cat rescue, stray cat, adoption

## Abstract

**Simple Summary:**

Animal welfare organizations accept large numbers of cats with no known history. Because shelters are often highly stressful environments for cats, it is likely to be difficult to differentiate a frightened cat that is socialized to humans from a feral cat that is not. However, this distinction can help channel cats into appropriate dispositions. We conducted structured assessments to measure various behaviors and their potential to distinguish socialization levels. Our results show that a specific set of behaviors are only exhibited by more socialized cats. Many cats needed time to adjust to the shelter-type setting to show these socialized behaviors.

**Abstract:**

Animal welfare organizations routinely accept large numbers of cats with unknown histories, and whose backgrounds vary from well-socialized pets to cats that have had little or no contact with humans. Agencies are challenged with making the determination of socialization level in a highly stressful environment where cats are often too frightened to show typical behaviors. A variety of structured behavioral assessments were conducted in a shelter-like environment, from intake through a three day holding period, on cats from the full range of socialization as reported by their caregivers. Our results show that certain behaviors such as rubbing, playing, chirping, having the tail up or being at the front of the cage were found to be unique to More Socialized cats. While not all more socialized cats showed these behaviors, cats that did were socialized. Assessing the cats throughout the three day period was beneficial in eliciting key behaviors from shyer and more frightened cats. These results will be used in future work to develop an assessment tool to identify the socialization status of cats as a standardized guide for transparent and reliable disposition decisions and higher live release rates for cats in animal shelters.

## 1. Introduction

Many cats enter animal shelters each year in the United States [1,2]. At least half are free-roaming cats brought to the shelter by people who are not their owner and, therefore, these cats enter the shelter with no known behavioral or socialization history [3,4,5]. The manner in which an animal welfare organization determines the options available for an incoming cat depends heavily upon the health, behavior and apparent socialization level with people of that cat. Shelters are tasked with determining where along the socialization spectrum a cat falls, from well-socialized to feral (un-socialized). Making this determination more difficult is the fact that during the first several days or weeks in an animal shelter, cats are likely highly stressed and may not show their typical behaviors. Also, cats show a range of time needed to acclimate to the environment or “de-stress”. It has been found that there is a pronounced decrease in stress levels in cats over the first four days; however 4% of cats showed hardly any reduction in stress levels after two weeks [6]. Despite this, cats are not typically given much time to de-stress in the shelter environment. Shelters routinely make disposition decisions about cats entering their facility in a very short time span, on average within 24–72 hours [7]. 

Our definition of socialization for this project was focused on the cat’s level of comfort and familiarity with humans. Truly feral cats are too frightened of humans to be placed into a home as a companion animal [8]. Disposition options for such cats are typically neuter-vaccinate-return to where they were found (return-to-field) or euthanasia [7]. In addition, unsocialized cats held for extended and unnecessary periods of time experience severe stress and poor welfare, making prompt identification an important consideration for their well-being. Options for some shy yet more socialized cats might include behavioral rehabilitation in the shelter, foster/adoption into a quiet, patient home that can provide time, behavior modification training, and a stable environment for the cats to overcome their fear, or return-to-field after neuter programs. Cats who quickly display behaviors indicating that they are well accustomed to humans can be fast-tracked into the shelter’s adoption process. It is important to make an accurate determination of socialization so the appropriate disposition track is chosen. Incorrect identification of a cat’s socialization status could result in euthanasia or return-to-field of a frightened but socialized cat that would have benefited from time to show more socialized behaviors. 

Despite the importance of differentiating between more socialized but frightened cats from cats that are truly feral, there are currently no validated methods of assessing or categorizing cats upon intake to an animal shelter. The shelter setting places a variety of constraints on development and implementation of such an assessment. Shelter staff have limited time and variable levels of experience and training in cat behavior, so such an assessment would need to be as simple and as objective as possible. The assessment also should be designed to be performed in a consistent way with relatively little training, to minimize the possibility of staff injuries when handling cats with unknown backgrounds, and to limit transmission of contagious disease between cats. Finally, most research on cat behavior in shelters or shelter-like environments has centered only on indicators of stress [9,10] but not on behavioral indicators for socialization. Given this lack of existing data, development of an accurate and valid assessment tool would require examination of wide variety of behaviors and physical measures to identify those most indicative of socialization status.

This study is the second of a three-phase project with a goal of developing a reliable and valid assessment tool that shelter staff can use to differentiate cats by socialization status within three days after intake. The first phase evaluated the reliability and validity of the Cat Behavior and Background Survey used to determine how socialized cats were in their normal environment, which provided the level of socialization used in this phase [11]. We recognize that there is no established standard for determining a cat’s comfort with humans; however, a tool was needed to better understand how to identify socialized cats. The present manuscript serves to (1) describe the origin and administration of the structured assessments and measures used and their practicality in a shelter setting, (2) describe how the behavioral and physical measures changed in biologically important ways over time and by type of structured assessment (interaction) with the cat (for further analysis of these measures, see the third article in the series [12]), (3) examine which behaviors and physical measures are unique to More Socialized cats (based on their Cat Behavior and Background Survey results, see [11] for more detail) within three days after shelter intake to create a Behavior Checklist, and (4) validate the results of the Behavior Checklist.

## 2. Methods

### 2.1. Subjects

This study was approved by the ASPCA® Internal Review Board and was conducted from January through April 2012 at People for Animals Spay/Neuter Clinic and Rescue (PFA) in Hillside, New Jersey, United States. Participants (both owners and caregivers of cats) were recruited through professional networking, online advertisements on nj.com, banner ads on PFA’s website, articles about the study, an ASPCA member regional news alert, flyers, and phone conversations with callers to PFA. We encouraged the participation of owners and caregivers to bring in cats anywhere along the spectrum of socialization towards humans, from truly un-socialized ferals to well-socialized house pets. All cat owners, fosterers and caregivers signed an informed consent form permitting their cats to participate in the study and completed the Cat Behavior and Background Survey [11]. Participating owned and fostered cats must have been living in the home for at least one month; unowned, free-roaming cats had to have been seen regularly by the caregiver for at least one month. Up to four cats per owner/caregiver were eligible to participate, with the limit raised to five if at least one of the cats was a poorly socialized cat. 

Cats eligible for participation were not visibly pregnant, nursing, nor obviously in heat, were between six months and ten years old by best estimate, and in good health. Cats could be excluded at any time during the study if, based on a visual assessment, they did not meet these criteria. Health status was visually assessed by a veterinarian twice a day for overt signs of illness including ocular or nasal discharge, squinting, sneezing, or severe lethargy, and confirmed by physical examination as necessary. Visual inspection was used for exclusions as it is the most commonly used method that shelters employ on a large scale. Any cats displaying signs of illness were excluded and returned early to their owner, fosterer or caregiver. To limit disease transmission, owned cats and unowned cats (rescue or outdoor free-roaming cats) were studied in alternate study replicates.

To encourage participation, several benefits were offered for the cats and owner/caregivers. All unaltered cats were spayed or neutered at no charge or a nominal fee. All cats received a free FVRCP (feline viral rhinotracheitis, calicivirus and panleukopenia virus) vaccination, and a topical flea/tick treatment.

We were aiming for 300 cats to provide relatively precise confidence intervals on the estimates for each measure. With 300 cats and 50% of cats with one response category, the 95% confidence interval was 0.5 (0.44–0.56). There were 302 cats believed to be eligible at the start of the study, but two cats were subsequently excluded from analysis due to late pregnancy or going into heat and one cat due to apparent painfulness, one due to severe lethargy, and one due to severe URI. Therefore, data from 297 cats were retained for analysis. 

The median number of cats in the study from each owner/caregiver was two (range 1 to 7). Four people brought in more than the five cat limit (median of six cats, range 6–7). A total of 161 owners/caregivers participated. 

There were 159 male cats (54%) and 138 female cats (46%). In general, sex was determined after the three day study period during the pre-surgical exam unless the cat was particularly friendly or visual identification was possible. Three males were cryptorchid and 12 females were early- to mid-term pregnant and/or lactating and one was in heat; all were included in this study since these conditions were not visually identified during the three day study period. The ages of the cats were estimated by the caregivers and by the veterinarian during the pre-surgical exam (five cats did not have an estimated age). Age range was estimated at six months to eight years, with a median of approximately one year of age. There were more owned/fostered (n = 161) than unowned cats (n = 136). 

In addition, a previous, similar study was included for validation of results. This previous study consisted of 250 cats seen from April through October, 2010 at the Humane Alliance Spay/Neuter Clinic in Asheville, North Carolina, United States (HA) using the same approval, recruitment and inclusion criteria as the PFA data described above. There were 119 male cats (48%) and 128 female cats (52%) with three unknown (the owners did not provide data on cats’ sex and because they indicated the cats were neutered they did not receive a pre-surgical examination). Age range was estimated at six months to eight years, with a median of approximately one year of age. There were similar numbers of owned/fostered (n = 124) and unowned cats (n = 126).

### 2.2. Intake and Housing Procedures

On Day 1 the cats were brought by their owners or caregivers to the study location in traps or carriers by 11 am. The owner/caregiver completed an informed consent form, the clinic’s standard surgery intake form, and a survey about the cat’s usual behavior and duration of ownership/care (Cat Behavior and Background Survey [11]). Carriers and traps were carried to a waiting area outside the study room and covered with a sheet to prevent contact between adjacent cats. Once all cats had arrived, each cat was brought into the study room in the order that they arrived, given vaccines, flea treatment, and a visual health exam, and placed in the appropriate numbered cage. For this process, the cats that arrived in carriers were taken out of the carrier, cats that arrived in trap were placed on a table 1 meter high and a trap divider was used to immobilize the cat.

To simulate a typical shelter environment, the study room contained 16 stainless steel holding cages. One bank of cages was arranged in two rows of six, one row on top of another. An additional four cages (two rows of two cages) sat perpendicular to the other bank of cages, with a visual barrier preventing the cats from seeing any other cats. The lower cages were elevated 10” off of the floor. Cages were 24” wide × 24” high × 29” deep, and included a 20” wide × 15” deep × 13” high Kuranda™ bed at the back of the cage. Each cage contained food and water bowls mounted on the cage door at the left front, a litter box at the right front of the cage, a plush bed at the back underneath the Kuranda™ bed, and a washcloth to cover the remaining cage floor. Water was offered *ad libitum*. Food was offered *ad libitum* 20 hours per day: 0.3 kg dry cat kibble topped with a heaping tablespoon of canned cat food and one moist cat treat placed on top of the food as an aid in determining if the cat ate or not. 

### 2.3. Structured Assessments and Measures

The structured assessment methods and the measures included here were chosen to encompass the wide variety currently in use to determine how well socialized cats are with people as well as those we deemed potentially predictive. We included those used by shelters, rescues and fostering stake-holders [7], as well as assessments found in published feline stress measures [6,9], feline behavior evaluations [13,14,15], flight and defense behavior in cats [16], written but unpublished shelter cat evaluations in use in five other animal shelters and rescues, and our previous work done at HA. We also added the Push with Rod to the Stroke with Rod assessment to elicit as much response from shy but socialized cats as possible. This assessment was stopped if the cats grew too distressed (based on the opinion of the observer).

All structured assessments and measures were pilot tested on 29 cats, then modified based on feedback from a research assistant and one of the authors (KMiller) who trained the research assistant. We originally included the Cat Stress Score [6] as a measure to include stress. This system has 11 body parts or activities that must be rated simultaneously. We found the scoring system too difficult and time consuming for our purposes since it depended on rating many body and head positions that often fell under different scores. To retain what appear to be important indicators of stress that were elements of the Cat Stress Score and which could be useful for our objectives, we broke down the components of this Score. We retained but sometimes simplified body position, tail position, head position, eye open or closed, ear position, vocalization and elements of the activity category. 

The final set of six structured assessments featured 46 behavioral, physical, or environmental measures. Table 1 describes the structured assessments and the order they were completed. The structured assessments were conducted over three days, with separate assessment periods occurring each morning and each afternoon, for a total of five time periods (PM-Day 1; AM-Day 2; PM-Day 2; AM-Day 3; PM-Day 3).

All structured assessments were conducted in-person by one observer (ND for the first 10 weeks, NT for the last 15 weeks at PFA or AM at HA). Each observer was trained by one author (KMiller) for a week to obtain consistent results and supervised periodically. Written descriptions of the assessments and measures were also provided to the observers. Any questions or issues related to the cats or assessments were promptly shared with the author supervising the project (KMiller) and resolved. The observer was aware if it was an owned or unowned cat week but did not have access to the Cat Behavior and Background Survey and was not present during intake to decrease bias. A GoPro™ video camera was mounted on a chest harness of the observers and all assessments were video recorded for reference as needed.

Once all cats had been through the intake process on the morning of Day 1, they were left undisturbed for one hour. The cats’ behavior was then assessed inside their cages in the afternoon of Day 1 from approximately 1 pm to 6 pm (PM-Day 1). Cats were fed during the final assessment of the day, Eating in Presence of Observer, after which they were left undisturbed in their cages during the night with the light off. On Days 2 and 3, assessments were performed from 8 am to 1 pm (AM-Day 2 and AM-Day 3), followed by removal of food bowls and spot cleaning/maintenance from 1pm to 2 pm by the observer, left one hour undisturbed, then further assessments from 3 pm to 6 pm (PM-Day 2 and PM-Day 3). A white noise machine was turned on in the morning and run all day during assessments to help with noises outside the room. Again, cats were fed during the last assessment of the day and left undisturbed all night. 

The order in which cats were observed in their cages was randomly decided for each AM and PM assessment period. The observer would complete all of the assessments in order for each cat, before proceeding to the next. All data was collected using an Acer™ or an iPad™ tablet using a spreadsheet in Microsoft Excel. 

**Table 1:** Description of the structured assessments and the order they were performed.

Assessment	Description	Duration
**Greet**	Observer stands 30 cm in front of cage, facing cat, holds out hand palm-up, and talks softly to cat for 15 seconds. After done, assess cage condition: evidence of eating, drinking, litter box use, and dishevelment of cage furnishings is noted.	15 seconds
**Crack cage door**	Observer stands 30 cm in front of cage facing cat, says, “Hi, Kitty!” in a friendly voice then places hand on door handle of cage, poised to open it, for 30 seconds. Door is then cracked open 2 cm for 3 seconds then immediately closed and latched.	30 seconds
**Novel object**	One of three different unfamiliar objects is hung on outside of the cage door: a pair of sunglasses, a clean litter box scoop, or a red plastic cup. Observer stands 60 cm in front of cage, at a 45 degree angle to cage front, not facing the cat or making direct eye contact.	30 seconds
**Interactive toy**	Observer stands 30 cm in front of cage, facing the front of cage, not facing the cat or making direct eye contact. A 30 cm long white cotton string attached to a rod was jiggled just inside the closed cage door to encourage play for 30 seconds. A new string is used for every cat.	30 seconds
**Touch with rod (includes Stroke with Rod and Push with Rod)**	Observer stands 30 cm in front of the trap or cage, facing cat, and slowly extends a 1 m long rubber-tipped rod toward cat through bars of trap. Rod’s movement is stopped 2.5 cm in front of cat's face for 5 seconds, to allow cat to sniff if s/he chooses. **Stroke with Rod**: Observer then attempts to stroke cat gently on cheek and side of neck. If cat avoids petting, observer attempts to pet cat for max 10 seconds before ending test. If cat can be petted, observer attempts to stroke cheek and neck for 10 seconds. Observer then pulls rod back to 2.5 cm in front of cat's face and pauses 5 seconds, then attempts to pet cat again in same way. **Push with Rod**: Then observer firmly and steadily pushes down and toward the back of the cage on cat’s shoulders with rod for 10 seconds. If the cat becomes too distressed, the test is ended. Rod cleaned with Trifectant between cats.	40 seconds
**Eating in the Presence of observer**	Only performed once each day. On Day 1 it is unknown when cat has eaten last but food has not been available since intake. On Days 2 and 3, food was removed mid-day 4 hours prior to this assessment. Observer quietly places food bowl inside cage. Bowl contains dry food, canned food, and one semi-moist cat treat. Observer then stands quietly 1 m away, not facing the cat or making direct eye contact and observes cat’s behavior. Observer is positioned centrally in front of bank of cages to observe up to 8 cats at one time.	10 minutes

On Day 4 all unaltered cats underwent sterilization surgery and received a rabies vaccination; cats that were known to be altered already were returned to their owners/caregivers on Day 4. While cats were anesthetized for surgery, a veterinarian or the observer noted their body condition, reproductive status (neutered or intact), estimated age, tooth/gum condition, and any abnormalities found. Cats recovered post-surgery in the trap or carrier they arrived in covered with a sheet, then were returned to their owners/caregivers on the evening of Day 4. The Appendix Table A1 includes a table that summarizes the behavioral, physical and environmental measures that were taken during each structured assessment.

### 2.4. Socialization Scores and Categories

Each owner or caregiver completed the Cat Behavior and Background Survey for each cat. Overall Socialization Score was calculated by taking the median score of 11 behavioral ratings from the Cat Behavior and Background Survey, as previously described [11]. Socialization Scores then were dichotomized into one of two Socialization Categories for statistical analyses. The cut-point was based on the natural distribution of the scores. The dichotomous Socialization Categories created were “Less Socialized” (Socialization Scores of 0 to 3), which were those at the very low end of the socialization scale, and “More Socialized” (Socialization Scores of 4 to 10), which were those in the middle to high end of the socialization scale.

### 2.5. Development of the Behavior Checklist Using PFA Data

To begin the process of developing an effective assessment tool, we evaluated the structured behavior assessments to gauge their potential to differentiate socialization levels of cats. A number of affiliative and interactive behaviors were exhibited primarily or exclusively by More Socialized cats and were identified for their potential to separate More Socialized from Less Socialized cats. Only the total number of affiliative behaviors (rub, knead, touch, play, sniff, roll, reach) were entered into the data sheet. One author (ND) reviewed all the videos, determined and then entered into the data sheet which specific affiliative behaviors were shown by each cat in each assessment. Based on this, a Behavior Checklist was developed where these affiliative/interactive behaviors were divided into categories “Strong” and “Weak” (Table 2). Strong behaviors were clearly responses of cats accustomed to humans based on the data and the authors’ experiences and Weak behaviors were shown by Less Socialized cats on rare occasions but which, when seen repeatedly, were likely indicative of socialization based on the authors’ experiences and the data. The Strong behaviors included those that involved contact with the observer such as rub, touch observer, and play, as well as behaviors such as kneading and chirping vocalizations. Weak behaviors included behaviors such as sniffing or “still moving” at the end of the assessment. We considered one Strong behavior and/or four Weak behaviors to classify a cat as More Socialized and these numbers were determined and confirmed by examining the Socialization Category and reviewing video recordings. Consensus by a team of the authors (EW, LG, MS, ND) that a cat was in fact behaving as a More Socialized cat on the videos was used as final criterion for confirmation of which behaviors belonged as Strong or Weak on the Behavior Checklist.

We also attempted to follow-up with a set of these cats that had different conclusions based on the Socialization Category and the Behavior Checklist. We introduced the conversation via telephone or email with the caregiver by indicating that we were following up a set of cats that were very unsocialized based on their survey. If they responded that they remembered the cat and still interacted with the cat, we then asked if the cat’s behavior had changed at all since the study. If they said the behavior had changed, we emailed them the Cat Behavior and Background Survey questions to answer again.

**Table 2:** Behavior Checklist describing affiliative behaviors displayed by cats divided into Strong and Weak behaviors.

Assessment	Strong behaviors	Weak behaviors
Greet	Rub Knead Touch Play At front of cage at any time	Sniff Roll Reach Approach front of cage at any time
Crack Cage Door	Chirp at any time	Sniff
	Rub	Roll
	Knead	Reach
	Touch	Approach front of cage at any time
	Play	Yawn at any time
	At front of cage at any time	Groom/shake at any time
	Tail position is up in the air at end of assessment	Body position at end of assessment is stand/move
		Location at end of assessment is still moving
Novel Object Interactive Toy	Chirp at any time Rub Knead Touch Play At front of cage at any time Tail position is up in the air at end of assessment	Sniff Roll Reach Approach front of cage at any time Yawn at any time Groom/shake at any time Body position at end of assessment is stand/move Location at end of assessment is still moving
Touch with Rod	Chirp at any time Rub (stroke and push each count) Knead (stroke and push each count) Touch (stroke and push each count) Play (stroke and push each count) At front of cage at any time	Sniff (stroke and push each count) Roll (stroke and push each count) Reach (stroke and push each count) Approach front of cage at any time Yawn at any time Groom/shake at any time

### 2.6. Statistical Analysis

For each measure, the number and percentage for More Socialized and Less Socialized cats based on the Socialization Category were calculated. Measures were then statistically analyzed with the cats’ Socialization Category using Fisher exact test. Due to the large number of statistical analyses conducted on the same data set, the p-values should not be considered to be accurate for the overall study error rate. The p-values were used only as guidance on which measures to consider for the Behavior Checklist and the final Checklist was developed as described in Section 2.5. Cat Under/Behind Stuff and Cat Turned Away as responses which indicated that the response for the measure could not be observed were kept as separate categories in the analyses to avoid having missing data. The patterns of what time period the cats exhibited Strong and Weak affiliative/interactive behaviors were also summarized with the number and percentage. Spearman rank sum correlation coefficients and their 95% confidence intervals were calculated for Strong and Weak behaviors and the Socialization Score. Statistical analyses were conducted using StataSE 12 (64 bit) (StataCorp LP, College Station, TX, USA) and graphed in Microsoft Excel.

**Table 3:** Measures that were significantly associated with Socialization Category (Less and More Socialized) and used in the Behavior Checklist.

Measure	PM-Day 1									AM-Day 2									PM-Day 2									AM-Day 3									PM-Day 3								
	Less social (n = 85)			More social (n = 212)						Less social (n = 85)			More social (n = 212)						Less social (n = 85)			More social (n = 208)						Less social (n = 85)			More social (n = 206)						Less social (n = 84)			More social (n = 205)					
	N (%)			N (%)			p-value			N (%)			N (%)			p-value			N (%)			N (%)			p-value			N (%)			N (%)			p-value			N (%)			N (%)			p-value		
Greet																																													
**Chirp at any time**							0.45									0.001									0.006									0.004									0.003		
Yes	1(1)			7 (3)						0			20 (9)						1 (1)			21 (10)						1 (1)			23 (11)						2 (2)			28 (14)					
**Strong affiliative behaviors**							0.3									0.001									0.001									0.001									0.001		
Yes	0			11 (5)						0			29 (14)						2 (2)			32 (15)						0			71 (34)						2 (2)			64 (31)					
**Weak affiliative behaviors**							0.5									0.001									0.001									0.001									0.001		
Yes	0			39 (18)						2 (2)			91 (43)						2 (2)			75 (36)						2 (2)			160 (51)						4 (5)			96 (47)					
**Approach at any time**							0.001									0.001									0.001									0.001									0.001		
At Front	0			12 (6)						0			17 (8)						2 (2)			14 (7)						2 (2)			32 (16)						0			10 (7)					
Yes ^a^	1 (1)			19 (9)						1 (1)			44 (21)						1 (1)			49 (24)						0			55 (27)						1 (1)			27 (18)					
Crack Cage Door																																													
**Chirp at any time**							1.0									1.0									0.6									0.04									0.3		
Yes	0			2 (1)						0			2 (1)						0			3 (1)						0			10 (5)			5			1 (1)			8 (4)					
**Strong affiliative behaviors**							0.002									0.001									0.001									0.001									0.001		
Yes	0			18 (8)						1 (1)			49 (23)						2 (2)			52 (25)						0			100 (49)			49			6 (7)			94 (46)					
**Weak affiliative behaviors**							0.001									0.001									0.001									0.001									0.001		
Yes	0			31 (15)						2 (2)			63 (30)						2 (2)			70 (34)						1 (1)			102 (50)						4 (5)			86 (42)					
**Approach at any time**							0.002									0.001									0.001									0.001									0.001		
At Front	0			13 (6)						0			30 (14)						1 (1)			27 (13)						1 (1)			50 (24)						2 (2)			51 (25)					
Yes ^b^	0			11 (5)						1 (1)			28 (13)						0			24 (12)						1 (1)			31 (15)						1 (1)			29 (11)					
**Yawn at any time**							0.4									0.5									0.004									0.7									0.25		
Yes	0			6 (3)						1 (1)			6 (3)						0			17 (8)						0			5 (2)						2 (2)			13 (6)					
**Groom/ shake body at any time**							0.6									0.03									0.004									0.03									0.09		
Yes ^b^	0			4 (2)						1 (1)			17 (8)						0			16 (8)						0			12 (6)						3 (4)			20 (10)					
**Body Position at end**							0.006									0.001									0.001									0.001									0.001		
ventral/ crouch/crawl	38 (45)			53 (25)						34 (40)			46 (22)						24 (28)			30 (14)						24 (28)			30 (15)						19 (22)			20 (10)					
standing/ moving ^b^	0			7 (3)						0			36 (17)						1 (1)			29 (14)						1 (1)			52 (25)						2 (2)			46 (22)					
lay on side/half side	45 (53)			135 (63)						45 (53)			111 (52)						58 (67)			131 (63)						53 (62)			101 (49)						61 (72)			119 (58)					
**Tail position at end**							0.42									0.001									0.001									0.001									0.001		
Up	0			4 (2)						0			21 (10)						1 (1)			24 (12)						0			43 (21)						1 (1)			38 (19)					
Down, back	2 (2)			15 (7)						1 (1)			36 (17)						1 (10			26 (13)						6 (7)			32 (16)						0			24 (12)					
Loose wrap	10 (12)			27 (13)						9 (11)			36 (17)						13 (15)			41 (20)						9 (11)			33 (16)						9 (11)			40 (20)					
Tight wrap	57 (67)			131 (62)						52 (61)			97 (46)						58 (67)			93 (45)						60 (71)			83 (40)						61 (72)			85 (42)					
**Location in cage at end**							0.42									0.03									0.8									0.01									0.001		
Still moving	0			0						0			8 (4)						1 (1)			7 (3)						0			11 (5)						0			13 (6)					
Shelf	16 (19)			28 (13)						48 (57)			81 (38)						41 (48)			90 (43)						54 (64)			90 (44)						52 (61)			87 (42)					
Bed	40 (47)			114 (54)						15 (18)			61 (29)						22 (27)			60 (29)						13 (15)			36 (18)						13 (18)			39 (19)					
Novel Object																																													
**Strong affiliative behaviors**							0.6									0.002									0.001									0.001									0.001		
Yes	0			4 (2)						0			18 (8)						2 (2)			34 (16)						0			74 (36)						2 (2)			66 (32)					
**Weak affiliative behaviors**							0.01									0.001									0.001									0.001									0.001		
Yes	0			14 (7)						1 (1)			27 (13)						2 (2)			48 (23)						0			79 (38)						4 (5)			70 (34)					
**Approach at any time**							0.04									0.001									0.001									0.001									0.001		
At Front	0			12 (6)						0			22 (10)						1 (1)			24 (12)						2 (2)			53 (26)						1 (1)			52 (25)					
Yes	0			3 (1)						0			13 (6)						0			10 (5)						0			17 (8)						2 (2)			14 (7)					
**Groom/shake body at any time**							0.6									0.05									0.001									0.06									0.2		
Yes	0			3 (1)						1 (1)			16 (8)						0			21 (10)						0			9 (4)						1 (1)			10 (5)					
**Body Position at end**							0.4									0.002									0.001									0.001									0.001		
ventral/crouch/crawl	34 (40)			60 (28)						33 (39)			43 (20)						28 (33)			32 (15)						19 (23)			29 (14)						19 (23)			20 (10)					
standing/moving	0			6 (3)						0			22 (10)						1 (1)			17 (8)						1 (1)			38 (18)						1 (1)			24 (12)					
lay on side/half side	48 (57)			135 (64)						45 (53)			126 (59)						54 (64)			132 (64)						57 (67)			106 (51)						60 (71)			126 (62)					
**Tail position at end**							0.6									0.001									0.001									0.001									0.001		
Up	0			3 (1)						0			7 (3)						1 (1)			10 (5)						0			20 (10)						1 (1)			16 (8)					
Down, back	2 (2)			13 (6)						1 (1)			34 (16)						1 (1)			29 (14)						3 (4)			54 (26)						1 (1)			30 (15)					
Loose wrap	9 (11)			24 (11)						9 (11)			41 (19)						11 (13)			43 (21)						12 (14)			34 (17)						6 (7)			49 (24)					
Tight wrap	59 (69)			139 (66)						53 (62)			101 (48)						60 (71)			102 (49)						55 (65)			84 (41)						62 (74)			94 (46)					
**Location in cage at end**							0.4									0.2									0.2									0.001									0.001		
Still moving ^c^	0			1 (1)						0			2 (1)						0			1 (1)						0			4 (2)						1 (1)			2 (1)					
Shelf	16 (19)			32 (15)						47 (55)			88 (42)						41 (48)			89 (43)						55 (65)			84 (41)						53 (63)			87 (42)					
Bed	39 (46)			119 (56)						16 (19)			64 (30)						19 (22)			66 (32)						13 (15)			40 (19)						13 (15)			46 (22)					
Interactive Toy																																													
**Strong affiliative behaviors**							0.004									0.001									0.001									0.001									0.001		
Yes	0			17 (8)						0			51 (24)						2 (2)			58 (28)						4 (5)			110 (53)						6 (7)			75 (37)					
**Weak affiliative behaviors**							0.001									0.001									0.001									0.001									0.001		
Yes ^d^	0			20 (9)						1 (1)			60 (28)						2 (2)			60 (29)						2 (2)			89 (43)						5 (6)			72 (35)					
**Approach at any time**							0.005									0.001									0.001									0.001									0.001		
At Front	0			12 (6)						0			23 (11)						1 (1)			25 (12)						2 (2)			50 (24)						3 (4)			45 (22)					
Yes ^d^	0			9 (4)						0			18 (8)						0			23 (11)						0			24 (12)						2 (2)			25 (12)					
**Body Position at end**							0.2									0.02									0.001									0.001									0.001		
ventral/ crouch/crawl	34 (40)			57 (27)						34 (40)			56 (26)						33 (39)			31 (15)						21 (25)			36 (18)						19 (23)			60 (15)					
standing/ moving	0			4 (2)						0			16 (7)						0			21 (10)						1			27 (13)						1 (1)			20 (10)					
lay on side/half side	49 (58)			139 (66)						44 (52)			108 (51)						50 (59)			130 (63)						54 (64)			97 (47)						58 (69)			109 (53)					
**Tail position at end**							0.6									0.001									0.001									0.001									0.001		
Up	0			0						0			2 (1)						0			4 (2)						0			7 (3)						0			3 (1)					
Down, back	2 (2)			8 (4)						0			45 (21)						2 (2)			49 (24)						3 (4)			60 (29)						3 (4)			47 (23)					
Loose wrap	9 (11)			34 (16)						11 (13)			42 (20)						11 (13)			40 (19)						12 (14)			37 (18)						6 (7)			55 (27)					
Tight wrap	60 (71)			136 (64)						52 (62)			95 (45)						58 (68)			102 (49)						56 (67)			87 (42)						61 (73)			58 (42)					
**Location in cage at end**							0.6									0.3									0.6									0.002									0.009		
Still moving	0			0						0			1 (1)						0			0						0			0						0			0					
Shelf	15 (18)			35 (17)						47 (55)			89 (42)						41 (48)			92 (44)						53 (63)			81 (81)						52 (62)			93 (45)					
Bed	41 (48)			116 (55)						17 (20)			62 (29)						20 (24)			57 (27)						13 (15)			40 (20)						14 (17)			42 (21)					
Touch with Rod																																													
During stroking with rod																																													
**Strong affiliative behaviors**							0.1									0.001									0.001									0.001									0.001		
Yes	0			8 (4)						1 (1)			43 (20)						2 (2)			49 (24)						2 (2)			93 (45)						4 (5)			86 (42)					
**Weak affiliative behaviors**							0.001									0.001									0.001									0.001									0.001		
Yes	10 (12)			69 (33)						9 (11)			90 (42)						6 (7)			90 (43)						12 (14)			124 (60)						6 (7)			51 (25)			25		
During push with rod																																													
**Strong affiliative behaviors**							1.0									0.001									0.001									0.001									0.001		
Yes	0			1 (1)						0			25 (12)						0			25 (12)						0			57 (28)						3 (4)			57 (29)			29		
**Weak affiliative behaviors ^e^**							0.001									0.001									0.001									0.001									0.001		
Yes	2 (2)			35 (17)						4 (5)			69 (33)						2 (2)			66 (32)						9 (11)			86 (42)						8 (10)			79 (39)					
**Approach at any time**				0.3												0.001									0.001									0.001									0.001		
At Front	0			12 (6)						0			24 (11)						1 (1)			20 (10)						1 (1)			37 (18)						0			36 (18)					
Yese	1 (1)			7 (3)						3 (4)			23 (11)						1 (1)			29 (14)						1 (1)			38 (18)						6 (7)			34 (17)					

^a^ 2 Less Socialized cats showed Weak behaviors: 1 approach PM-Day 1, 1 sniff AM-Day 3; ^b^ 3 Less Socialized cats showed Weak behaviors: 1 approach PM-Day 3, 1 groom/shake PM-Day 3, 1 stand/move PM-Day 3; ^c^ 1 Less Socialized cats showed Weak behaviors: still moving at end of assessment PM-Day 3; ^d^ 8 Less Socialized cats showed Weak behaviors: 1 groom/shake PM-Day 2, 1 yawn PM-Day 2, 1 sniff PM-Day 2, 1 approach PM-Day 3, 1 yawn PM-Day 3; groom/shake and yawn not shown. ^e^ 10 Less Socialized cats showed Weak behaviors: 1 approach PM-Day 1, 1 groom/shake PM-Day 1, 1 approach AM-Day 3, 1 groom/shake AM-Day 3, 2 groom/shake PM-Day 3, 3 yawn PM-Day 3, 1 roll PM-Day 3; groom/shake and yawn not shown.

## 3. Results

### 3.1. Structured Assessment Evaluations

From the Behavior Checklist, the frequencies and percentages of the Strong and Weak behaviors and their associations with Socialization Category are summarized in Table 3. Only those measures which were used in the Behavior Checklist were included in Table 3. There were 66 of 212 More Socialized cats (31%) who did not demonstrate one or more Strong or four or more Weak behaviors and were therefore not considered to be socialized based on the Behavior Checklist. Cats classified as Less Socialized by Socialization Category regardless of the results of the Behavior Checklist were included as Less Socialized in Table 3 to demonstrate the strong association between Socialization Category and Strong and Weak behaviors.

**Figure 1:**
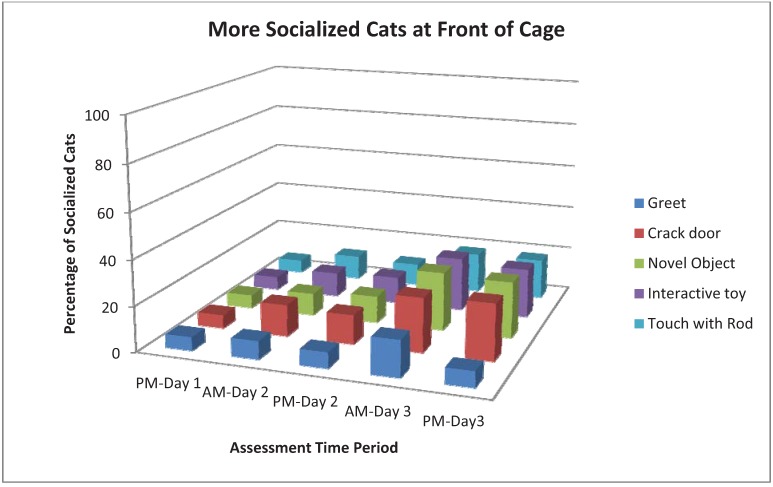
Percentage of More Socialized cats at front of cage during different assessments and across different time periods.

**Figure 2:**
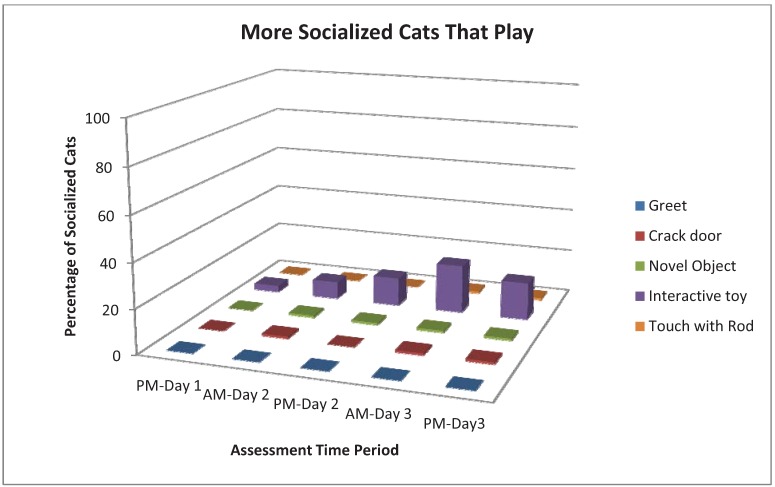
Percentage of More Socialized cats that played during different assessments and across different time periods.

**Figure 3:**
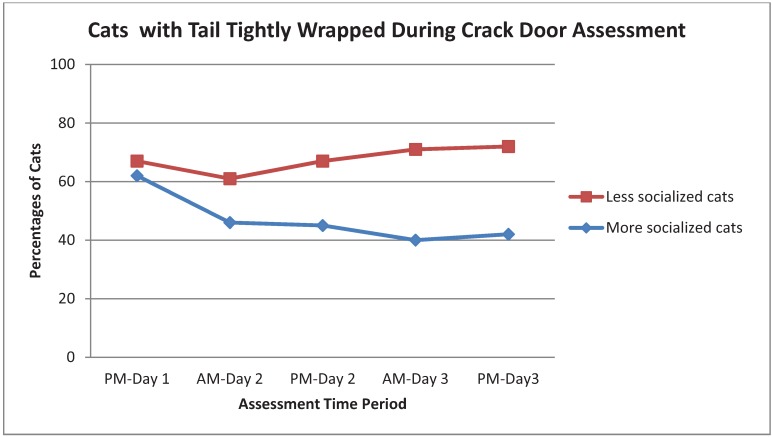
Percentage of More Socialized and Less Socialized cats with tails tightly wrapped during the Crack Cage Door assessment across different time periods.

**Figure 4:**
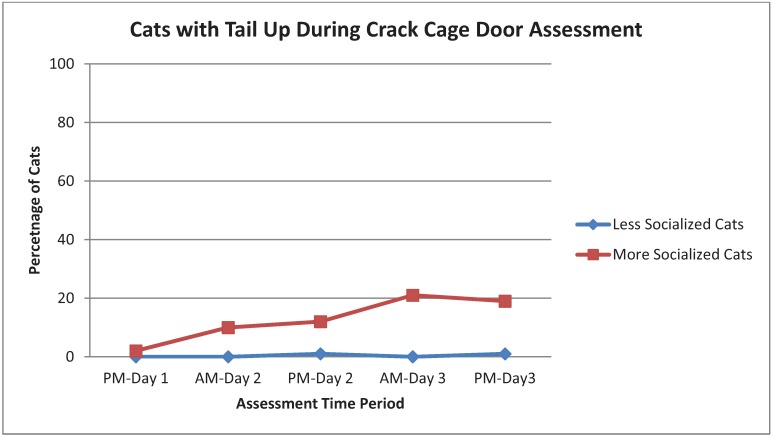
Percentage of More Socialized and Less Socialized cats with tails up during the Crack Cage Door assessment across different time periods.

As shown in Table 3, Strong behaviors were chirp, rub, knead, touch, play, being at the front of the cage and tail up. The incidence of chirping in More Socialized cats increased with time, especially during the Greet assessment. Cats that rubbed, kneaded or touched tended to do more of those behaviors with time. However, the assessments which evoked the most responses for Strong or Weak behaviors varied by behavior. For example, Crack Cage Door resulted in more rub and knead responses, and Interactive Toy evoked more touch and play. Being at the front of the cage was most common with Crack Cage Door, Novel Object and Interactive Toy and tended to increase through AM-Day 3 (Figure 1). Play was most common during Interactive Toy (Figure 2). More Socialized cats tended to gradually decrease the frequency of tightly wrapped tails (Figure 3) and crouched body position with time while Less Socialized cats continue to frequently display these behaviors. Tail up was most common during the Crack Door assessment, tended to increase with time and was almost exclusively shown by More Socialized cats (Figure 4). 

The patterns of Strong behaviors exhibited across time indicated that more than half (55%) of the More Socialized cats did not display any of these behaviors on Day 1. Seventeen percent of More Socialized cats didn’t show any Strong behavior until Day 3 while 15% of cats showed them for all time periods. However, 15% of More Socialized cats only showed Strong behaviors during a single time period. 

There were 11 cats (13%) who were considered to be Less Socialized based on the Cat Behavior and Background Survey who still exhibited one or more Strong behaviors in the assessments (as reviewed on video post-assessment). Of these 11 cats, there were six cats with more than one Strong behavior (2 to 22 behaviors). We were able to obtain follow-up with the caregivers of five of these cats; all caregivers reported substantial increases in Socialization Score based on questions in the Cat Behavior and Background Survey. There were two cats with only one Strong behavior, one of whom also showed three Weak behaviors (but we were not able to reach these cats’ caregivers). Three cats were classified as More Socialized based on Weak behaviors only (four to five Weak behaviors). We were able to reach one of those cats’ caregivers and determined that one cat had been adopted and was reported to be “doing well”. 

**Figure 5:**
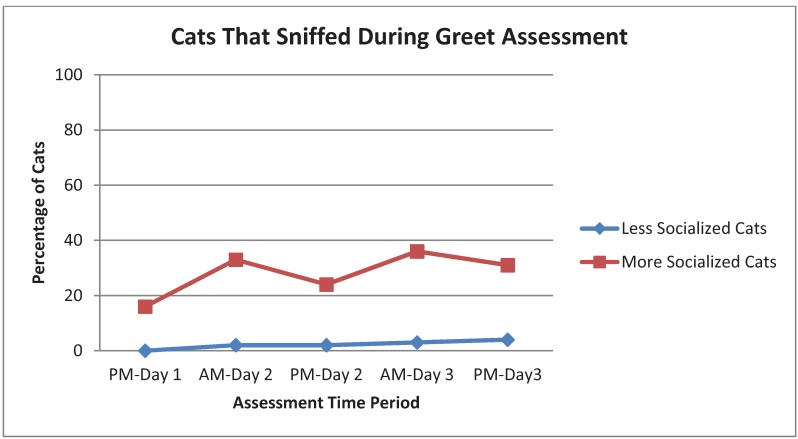
Percentage of More Socialized and Less Socialized cats that sniffed during the Greet assessment across different time periods.

Among the Weak behaviors (sniff, roll, reach, approach front of cage, yawn, groom, standing or moving and still moving at the end of the assessment), there were a variety of patterns across time periods and assessments. Sniff by More Socialized cats was common in all assessments but no consistent pattern of change across time periods was apparent (Figure 5). Rolling was most common during the first two assessments (Greet and Crack Cage Door) and tended to become more frequent across time periods. Reach toward observer or toy was most common by far with Interactive Toy and tended to peak in frequency during the AM-Day 3 time period. Approach and Body Standing or Moving (from Body Position at end of assessment) were most common for the Greet assessment and peaked at AM-Day 3. Yawning was most common in the Crack Cage Door assessment and the PM time periods. The pattern for Groom was unclear except for being much more common among More Socialized cats. Very few cats were Still Moving at the end of assessment but it was seen primarily in More Socialized cats during the Crack Cage Door assessment. 

The correlation between the Socialization Score and the total number of Strong behaviors was 0.54 (0.46–0.62), and the correlation between the Socialization Score and the total number of Weak behaviors was 0.57 (0.48–0.64). The correlation between Socialization Score and the sum of the Weak behaviors/4 plus the sum of the Strong behaviors was 0.75 (0.69–0.79).

The patterns of Weak behaviors exhibited across time indicated that more than half (51%) of the More Socialized cats did not display any of these behaviors on Day 1. Of the More Socialized cats, 16% did not show four or more Weak behaviors until Day 3. 

There were 32 cats (38%) categorized as Less Socialized who showed fewer than four Weak behaviors and no Strong behaviors and were not considered socialized based on the Behavior Checklist. Each cat only showed one or two Weak behaviors. See Table 3 for details. 

### 3.2. Validation of Behavior Checklist with Previous HA Data

In order to assess the validity of the Behavior Checklist, we used data from the previous study conducted at HA. There were four differences in the assessments for the HA data set compared to the PFA data set: (1) the Novel Object was only done during the PM time periods and the Interactive Toy only during the AM time periods; (2) each assessment was done with all cats and then the next assessment was done with all cats and so on (so all cats had the Greet assessment done first, whereas at PFA each cat had all six assessments done before moving on to the next cat). This sequence was changed at PFA to provide a longer and more consistent observer interaction to try to elicit as much response in the shy cats as possible; (3) there was a $10 incentive at a pet supply store added the third month for Less Socialized cats; (4) all specific affiliative behaviors during the assessments were individually entered into the data set which was not done at PFA. Several additional measures were used at HA but all measures in the current study were included in this validation data set.

The Behavior Checklist was applied to this HA data set to confirm results. Videos of these cats scored as Less Socialized by their caregivers but socialized by the Behavior Checklist were reviewed by one author (ND) and shared with the team to confirm socialization status by expert opinion as described in the Methods section.

Using the Behavior Checklist, the HA data set had 28 cats that were categorized as Less Socialized by Socialization Category but socialized by the Behavior Checklist. One of these cats had 12 Weak behaviors, four cats had only one Strong behavior and fewer than four Weak behaviors and two cats had only one Strong behavior and four or more Weak behaviors. The remaining 21 cats all had one or more Strong behaviors and/or four or more Weak behaviors. All videos were reviewed and cats were determined to be More Socialized. 

The overall correlation for all cats in the HA data set between Socialization Score and total Strong behaviors was 0.49 (0.39–0.58) and for total Weak behaviors 0.51 (0.42–0.60), and for both Strong and Weak 0.85 (0.81–0.88).

## 4. Discussion

This study was the second part (see [11,12] for parts 1 and 3) of a three-phase project towards the development of a valid and feasible assessment tool for determining the level of socialization of cats within three days of entering an animal shelter. To our knowledge, this is the first time that so many measures of basic appearance, behaviors and change in behavior over time in cats immediately after intake to a shelter-like setting have been scientifically collected and reported. In addition, this study focused on identifying those more objective measures which could indicate the socialization of cats with humans rather than general stress for cats in a shelter setting. Some of the results are for the most part, not surprising. For example, cats that made any movement toward the front of the cage were much more likely to be More Socialized. More importantly, we found that being at the front one third of the cage during an assessment was unique to More Socialized cats.

A very useful finding was cats that were socialized and comfortable with humans displayed unique behaviors which was very useful in beginning the process of identifying More Socialized cats. These behaviors were chirp, rub, knead, touch, play, being at the front of the cage at any time during an assessment and having a tail position of up in the air. When seen four or more times during the three days, additional behaviors unique to More Socialized cats were sniff, roll, reach, approach front of cage, yawn, and groom (all at any time during the assessment), as well as whether the cat was standing, moving or “still moving” at the end of an assessment.

In addition, the numbers of Strong and Weak behaviors were moderately well correlated with the Socialization Score of the cats. The combination of the numbers of Strong and Weak behaviors (where four Weak behaviors counted the same as one Strong behavior) showed much stronger correlations for both the PFA and HA data sets. This also supports the content of Behavior Checklist as those behaviors which are predictive of Socialization Category. The Behavior Checklist also could possibly be used to help decide how socialized a cat was, not just whether or not a cat was categorized as More Socialized or Less Socialized.

The authors’ review of the videos of the 11 cats that were scored as Less Socialized by the Cat Behavior and Background Survey but socialized by the Behavior Checklist confirmed that these cats’ behaviors, when viewed holistically, were very consistent with those of More Socialized cats. One possible reason for this disagreement between the Cat Behavior and Background Survey and behavior is that the caregivers had issues with understanding the survey, or that they misjudged the cat’s behavior. There was moderate to strong reliability and validity of the Cat Behavior and Background Survey [11] but there is certainly a possibility of error from that source. Another reason could be that the cats behaved differently in a caged environment without the option to run away or hide. In addition, type of interactions with the cats in the form of the assessment (*i.e.*, playing with a toy, touching with a rod) was different in the shelter-type environment compared to what likely occurred in the cat’s usual environment. When we were able to follow-up with some caregivers of these cats, all of the caregivers confirmed an increase in socialization based on the same questions from the Cat Behavior and Background Survey. We cannot rule out some bias on the part of the caregivers;, however the original Socialization Score from their survey was not shared with the caregivers. At HA, there was an added incentive for caregivers to bring in Less Socialized cats which could have influenced the number of cats who were Less Socialized by the Socialization Category but socialized by the Behavior Checklist.

The Behavior Checklist includes behaviors demonstrated to be unique to More Socialized cats in both the PFA and HA data sets. Therefore, the Behavior Checklist can identify More Socialized cats who exhibit these behaviors. However, the Behavior Checklist does not identify all More Socialized cats. We believe that this is due to varying levels of the cat’s boldness or outgoing personality as well as the cat’s need for social contact with humans [13]. Cats that are very valiant are likely to adapt to the novelty of a shelter environment more quickly and show their true socialization. This is supported by the finding that some More Socialized cats showed play behaviors even in the first afternoon (see Figure 2). Therefore, it is likely to be difficult to know just how soon a given cat will show playful behaviors. Cats that are more aloof and need much less social interaction with humans are likely to respond less often with affiliative behaviors. We suggest that cats that are very shy and very aloof are those who are most difficult to identify and who will provide the lowest number of Behavior Checklist behaviors, even Weak ones.

We hypothesize that chirping, yawning, approaching the front of the cage, rolling and still moving at the end of the assessment were most common during the Greet assessment because it was the first interaction with the human for that time period. Chirping was separated from meowing or purring because the authors considered it to be a sound that only More Socialized cats would make in the presence of the human and the data supported this hypothesis. In addition, the data did not demonstrate that other types of vocalizations were exclusive to More Socialized cats.

A critical finding was that the cats did not all have consistent patterns for which time periods they exhibited Strong behaviors or four or more Weak behaviors from the Behavior Checklist. Given the need to make a decision about socialization quickly, it is crucial to recognize that over half of the More Socialized cats did not show any Strong or Weak behaviors on Day 1. Twelve percent of More Socialized cats did not show any Strong or four or more Weak behaviors until AM-Day 3 and a similar percentage only showed these behaviors during one of the five time periods. Therefore, the data indicates that cats need to be interacted with on multiple occasions to be able to capture key behaviors that are indicators of socialization. This means that assessing cats, even using the Behavior Checklist behaviors, on arrival to a shelter, in a trap or otherwise, may not provide a complete or accurate picture of the cat’s true socialization status. 

Providing cats with three days to adjust to the environment and continued interaction in the form of the structured assessments resulted in many changes in behavior. For example, More Socialized cats tended to decrease the frequency of tightly wrapped tails and crouched body position with time and an increase in frequency of standing and moving. Less Socialized cats continue to frequently display tight tail wrapping and a higher frequency of crouching than More Socialized cats. These changes suggested that More Socialized cats were acclimating and experiencing less stress over time, in agreement with previous research on stress in cats [6,9]. Affiliative behaviors also tended to increase over time, especially the Strong behaviors. These findings support the potential importance of giving cats time to adjust and interact with the observer before making disposition decisions. This is important because 27% of animal welfare stakeholders in a nationwide survey reported making decisions about cats’ socialization status within the first 24 hours after intake [7]. Cats in the study came from a range of settings from indoor only to outdoor only pets and outdoor only cats with a variety of levels of care that were not claimed as owned. Cats also came from two different states in different regions of the country. Although it is not possible to know precisely how well the sampled cat population reflected the shelter cat population in those regions, or in the U.S. in general, we believe that our varied recruiting strategies, the cat participation incentives, the information we gained from the survey and conversations with caregivers at intake, the range of methods caregivers used to transport the cats for the study (carriers, traps, wire crates, cardboard boxes) and the range of feline behaviors observed during the study, all support the likelihood that the study population was a good reflection of the shelter cat population.

## 5. Conclusions

In conclusion, the present article determined that there are a set of assessments that could be used in a shelter-type environment. In addition, a specific set of behaviors were unique to More Socialized cats and provide one option to begin to differentiate More and Less Socialized cats. We found that multiple assessments completed over a three day period were valuable since a number of behaviors changed in frequency over time or were only demonstrated during one time period or assessment. A single assessment early in the three day period will likely only identify the most bold and demonstrative of More Socialized cats. These results will inform the development of an additional tool to separate socialized but frightened cats from unsocialized cats.

## Appendix

**Table A1:** Measures Taken During Each Structured Assessment.

Measures	Response choices	Assessment
**Affiliative behavior** (throughout assessment) *Obvious “friendly” or “affectionate” behavior displayed by the cat (including but not limited to Knead, Sniff, Rub, Roll, Touch gently with paw, Play, Lean in) throughout the assessment*	None 1 2 or 3 More than 3	Greet Crack Cage Door Novel Object Interactive Toy
**Affiliative PUSHING** (throughout PUSHING) *Obvious “friendly” or “affectionate” behavior displayed by the cat (including but not limited to Knead, Sniff, Rub, Roll, Touch gently with paw, Play, Lean in) throughout the pushing portion of the rod assessment until the end*	None 1 2 or 3 More than 3	Touch with Rod
**Affiliative STROKING** (throughout assessment) *Obvious “friendly” or “affectionate” behavior displayed by the cat (including but not limited to Knead, Sniff, Rub, Roll, Touch gently with paw, Play, Lean in) during attempts to STROKE the cat with the rod until the PUSHING portion of the rod assessment*	None 1 2 or 3 More than 3	Touch with Rod
**Agitated Vocalization** (throughout assessment) *Obvious agitated vocal sounds includes but not limited to yowl, growl, hiss, and spit*	Yes No Unknown (Cat turned away) Unknown (Cat under/behind stuff)	Crack Cage Door Novel Object Interactive Toy Touch with Rod
**Alertness** (at end of assessment) *Cat’s apparent awareness of its surroundings*	Alert *(eyes open, cat is fully awake and aware of its surroundings)* Semi-alert *(eyes are closed/partly closed while head raised, cat is somewhat aware of its surroundings)* Unalert *(cat’s eyes are closed and head is down on its paws, bedding or cage floor in a resting position, cat is apparently unaware of surroundings)* Unknown (Cat turned away) Unknown (Cat under/behind stuff)	Crack Cage Door Novel Object Interactive Toy
**Approach** (throughout assessment) *Cage is divided into thirds – a majority of the cat’s body (does not have to include stepping toward) moves forward, toward another 1/3 of the cage*	Yes *(cat moved majority of body into a forward third of the cage)* No *(cat did not move a majority of it’s body forward, toward another third of the cage)* At front *(cat was already right at front third of the cage and could not step any closer)*	Greet Crack Cage Door Novel Object Interactive Toy
**Approach rod** (throughout assessment) *The cat's feet stepped closer to the rod, no matter where the rod is located in the cage*	Yes *(cat stepped toward rod)* No *(cat did not step toward rod)* At front *(cat was already right at the front of the cage and could not step any closer and remained there throughout the assessment)*	Touch with Rod
**Attention to object** (throughout assessment) *Cat is interested, focused, and looking directly at object*	None *(cat does not look at the object but its eyes are open)* ≥50% *(cat looks at the object for ≥50% of assessment time)* <50% (*cat looks at the object for <50% of assessment time)* Eyes closed *(eyes are closed throughout the assessment)* Unknown (Cat turned away) Unknown (Cat under/behind stuff)	Novel Object
**Attention to person** (throughout assessment) *Cat is interested, focused, and looking at the person, includes but is not limited to eye contact*	None *(cat does not look at the observer but its eyes are open)* ≥50% *(cat looks at the observer for ≥50% of assessment time )* <50% *(cat looks at the observer for <50% of assessment time)* Eyes closed *(eyes are closed throughout the assessment)* Unknown (Cat turned away) Unknown (Cat under/behind stuff)	Novel Object Interactive Toy Touch with Rod
**Attention to rod** (throughout assessment) *Cat is interested, focused, and looking directly at the rod*	None *(cat does not look at the rod but its eyes are open)* ≥50% *(cat looks at the rod for ≥50% of assessment time)* <50% *(cat looks at the rod for <50% of assessment time)* Eyes closed *(eyes are closed throughout the assessment)* Unknown (Cat turned away) Unknown (Cat under/behind stuff)	Touch with Rod
**Attention to toy** (throughout assessment) *Cat is interested, focused, and looking directly at the toy*	None *(cat does not look at the rod but its eyes are open)* ≥50% *(cat looks at the rod for ≥50% of assessment time)* <50% *(cat looks at the rod for <50% of assessment time)* Eyes closed *(eyes are closed throughout the assessment)* Unknown (Cat turned away) Unknown (Cat under/behind stuff)	Interactive Toy
**Bedding**	No/mild disrupt *(bedding still in place or minor disruptions made such as bowls turned over or litterbox moved)* Very disrupted *(in essence, the cat has “trashed” the cage. For example, litter is everywhere or box is turned over, cage walls are “painted” with litter, bed is bunched up or upside down, kuranda bed/shelf is moved or turned over, etc. It should not be borderline. Borderline cages would be considered “mild disruption.”)*	Greet
**Blinking** (throughout assessment) *Cat closes then immediately reopens its eyes*	None *(cat does not blink*) Blinks with eye contact *(cat blinks while making eye contact with observer)* Blinks without eye contact *(cat blinks without making eye contact with observer)*	Greet Crack Cage Door Novel Object Interactive Toy Touch with Rod
**Blinks at rod** (throughout assessment) *Cat directs blink towards rod during “Touch with Rod” assessment (not applicable to any other assessment)*	None *(cat does not blink*) Blinks with eye contact *(cat blinks while making eye contact with observer)* Blinks without eye contact *(cat blinks without making eye contact with observer)* Unknown (Cat turned away) Unknown (Cat under/behind stuff)	Touch with Rod
**Body** (at end of assessment) *Position of cat’s body at the end of assessment*	Lay on back *(dorsal portion of cat’s body is to the floor, head usually upside down)* Lay on side/half side *(all or part of dorsal portion of cat’s body is angled to the side and back 2 or all 4 of cat’s feet are laying beside the cat)* Ventral/crouch/crawl *(dorsal portion of cat’s body is facing up and all four paws are directly underneath the cat, flat on the floor. Cat’s belly is touching or almost touching the floor. Cat may be crawling in this crouched position)* Sit *(Cat’s rear end is on the floor, all four paws are on the floor directly under the cat with front legs extended and back legs bent)* Stand/move back horizontal *(cat is standing or moving with hind quarters at same height as shoulder blades)* Stand/move behind low *(cat is standing or moving with hind quarters lower than shoulder blades)* Unknown (Cat turned away) Unknown (Cat under/behind stuff)	Crack Cage Door Novel Object Interactive Toy
**Body movement** (maximum observed) *Cat’s shoulders move along horizontal or vertical plane*	Yes Yes frantic No	Crack Cage Door
**Chirp** (at any time) *Short, burst of sound, such as a “coo” or “chirp” not defined as a meow or purr*	Yes No Unknown (Cat turned away) Unknown (Cat under/behind stuff)	Greet Crack Cage Door Novel Object Interactive Toy Touch with Rod
**Defensive/Aggressive behavior** (at any time) *Obvious distress behavior displayed by the cat including but not limited to, Paw raise, Swat (or try to swat), Bite (or try to bite), or Lunge directed at person, object or toy*	Yes No Unknown (Cat turned away) Unknown (Cat under/behind stuff)	Crack Cage Door Novel Object Interactive Toy
**Defensive/Aggressive PUSHING** *Obvious distress behavior displayed by the cat including but not limited to, Paw raise, Swat (or try to swat), Bite (or try to bite), or Lunge during the PUSHING portion of the rod assessment until the end*	Yes No Unknown (Cat turned away) Unknown (Cat under/behind stuff)	Touch with Rod
**Defensive/Aggressive STROKING** *Obvious distress behavior displayed by the cat including but not limited to, Paw raise, Swat (or try to swat), Bite (or try to bite), or Lunge during attempts to stroke the cat with the rod until PUSHING portion of the assessment*	Yes No Unknown (Cat turned away) Unknown (Cat under/behind stuff)	Touch with Rod
**Does cat eat?** (at any time during) *Does cat eat when food bowl placed in cage and observer stands about 4 feet away*	No Yes Sniff only	Eat in Presence of the Observer
**Drink**	None *(cat apparently did not eat or drink)* Part *(cat ate or drank part of water)* All *(cat ate and/or drank all water)* ? *(observer cannot distinguish whether cat has drank)*	Greet
**Ears** (at end of assessment) *Position of ears at the end of assessment*	Forward *(alert or relaxed, open portion of the ears id facing forward)* Flat *(tops of cat’s ears are flat or level with its head)* Sideways *(open portion of ears are facing to the side of the cat’s head)* Back *(open portion of ears is facing backwards)* Ears not visible *(observer cannot distinguish ear position)* Unknown (Cat under/behind stuff)	Crack Cage Door Novel Object Interactive Toy
**Eat** (only applicable on AM-Day 2 and AM-Day 3)	None *(cat apparently did not eat)* Part *(cat ate or drank part of food)* All *(cat ate and/or drank all food)* ? *(observer cannot distinguish whether cat has eaten)*	Greet
**Eye contact** (throughout assessment) *Any amount of eye contact with observer*	None *(cat makes no eye contact with observer)* Repeated ≥50% *(cat makes eye contact more than once during assessment and total duration is ≥50% of the assessment time)* Repeated <50% *(cat makes eye contact more than once during assessment and total duration is <50% of the assessment time)* Single ≥50% *(cat makes eye contact once during assessment and total duration is ≥50% of the assessment time)* Single <50% *(cat gives eye contact once during assessment and total duration is ≥50% of the assessment time)* Eyes closed *(eyes are closed during the entire assessment)* Unknown (Cat turned away) Unknown (Cat under/behind stuff)	Greet Crack Cage Door
**Eye position** (at end of assessment) *How open cat’s eyes are at the end of the assessment*	Open *(eyes are fully open)* Part closed *(eyes are partly closed, but observer is still able to see part of the cat’s eyeball)* Closed *(eyes are shut so observer is unable to see the cat’s eyeball)* Open – close *(the cat’s eyes are transitioning between open and closed, as in a blink or falling asleep)* Unknown (Cat turned away) Unknown (Cat under/behind stuff)	Crack Cage Door Novel Object Interactive Toy Touch with Rod
**Feces in cage** (at beginning of assessment) *Distribution of feces in cage*	None Localized Diffuse Unknown	Greet
**Grooming/Shake body** (at any time) *Cat uses tongue to clean body and/or cat “shakes off” its entire body. Does not include only scratching or biting at body*	Yes No Unknown (Cat turned away) Unknown (Cat under/behind stuff)	Crack Cage Door Novel Object Interactive Toy Touch with Rod
**Head facing** (at end of assessment) *Direction cat's head was facing in relation to the front of the cage and observer*	Away Sideways Toward Unknown (Cat turned away) Unknown (Cat under/behind stuff)	Crack Cage Door Novel Object Interactive Toy Touch with Rod
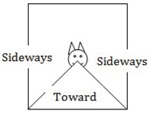
**Head position** (at end of assessment) *Position of center top of head in the cage*	Front third top third Back third	Greet Crack Cage Door Novel Object Interactive Toy Touch with Rod
**Head movement** (maximum observed) *Between the ears, center point of head moves horizontally or vertically*	Yes Yes frantic No	Crack Cage Door
**Lick nose/lips** (throughout assessment) *Any use of the tongue to touch the outside of the mouth, lips, or nose*	Yes No Unknown (Cat turned away) Unknown (Cat under/behind stuff)	Greet Crack Cage Door Novel Object Interactive Toy Touch with Rod
**Litter** (at beginning of assessment)	All in box *(all litter is contained in the litterbox)* <handful *(less than a handful of litter is outside of litterbox)* ≥handful *(greater than a handful of litter is outside of litterbox)* Dumped *(litterbox is upside down and/or litterbox is empty of litter)*	Greet
**Location** (at end of assessment) *A majority of the cat’s body is in this location of the cat at the end of the assessment*	Litterbox *(Gladwear pan used for litterbox)* Bed *(Toilet seat cover bed)* Shelf *(Kuranda bed shelf)* Under stuff *(under any bedding or litterbox) (does not include cat being under the kuranda bed shelf)* Anywhere else *(cat is anywhere else, other than litterbox, bed, shelf, or under stuff)* Still moving *(cat is moving between locations)*	Crack Cage Door Novel Object Interactive Toy
**Neutral Vocalization** (at any time) *Obvious neutral vocal sounds including meow and purr (does not include chirp)*	Yes No Unknown (Cat turned away) Unknown (Cat under/behind stuff)	Crack Cage Door Novel Object Interactive Toy Touch with Rod
**Sleep** (throughout assessment) *Whether cat is truly sleeping at any time during assessment*	Yes No Unknown (Cat turned away), Unknown (Cat under/behind stuff)	Crack Cage Door Novel Object Interactive Toy Touch with Rod
**Sniff rod 1** *Cat obviously orients his nose towards and obviously sniffs towards the rod within the “first sniff” five-second period before he’s petted with the rod the first time*	Yes No Unknown (Cat turned away) Unknown (Cat under/behind stuff)	Touch with Rod
**Sniff rod 2** *Cat obviously orients his nose towards and obviously sniffs towards the rod within the “second sniff” five-second period before he’s petted with the rod the second time*	Yes No Unknown (Cat turned away) Unknown (Cat under/behind stuff)	Touch with Rod
**Stiffen/Flinch/Lean away** (at any time) *Tightening the muscles OR flinching twitch of the skin when touched OR pulling the feet closer under the body and leaning away from person, object, or toy without moving the feet in the horizontal plane*	Yes No Unknown (Cat turned away) Unknown (Cat under/behind stuff)	Crack Cage Door Novel Object Interactive Toy
**Stiffen/flinch/lean away PUSHING** *Tightening the muscles OR flinching twitch of the skin when touched OR pulling the feet closer under the body and leaning away from the rod without moving the feet in the horizontal plane during the PUSHING portion of the rod assessment until the end*	Yes No Unknown (Cat turned away) Unknown (Cat under/behind stuff)	Touch with Rod
**Stiffen/flinch/lean away STROKING** *Tightening the muscles OR flinching twitch of the skin when touched OR pulling the feet closer under the body and leaning away from the rod without moving the feet in the horizontal plane during the STROKING portion of the rod assessment until PUSHING*	Yes No Unknown (Cat turned away) Unknown (Cat under/behind stuff)	Touch with Rod
**Stretch** (at any time) *Draw out, spread, or extend limbs and/or toes*	Yes No Unknown (Cat turned away) Unknown (Cat under/behind stuff)	Greet Crack Cage Door Novel Object Interactive Toy Touch with Rod
**Tail** (at end of assessment) *Position of tail at the end of assessment*	tight wrap *(tail is curled up tightly around the body, with no space between the tail and the body)* loose wrap *(tail is somewhat curled around the body but there is some space between the tail and the body)* down, back *(tail is down and behind the body up: the majority of the tail is held higher than the cat’s back)* wag/thump *(agitated wagging, swishing, or thumping of the tail on the cage floor)* ? Tail not visible *(observer cannot distinguish tail position)* Unknown (Cat under/behind stuff) *(cat is under or behind objects so observer cannot distinguish tail position)*	Crack Cage Door Novel Object Interactive Toy
**Urine in cage** (at beginning of assessment) *Distribution of urine in cage*	None Localized Diffuse Unknown	Greet
**Withdraw** (at any time) *Cage is divided into thirds – a majority of the cat’s body (does not have to include stepping toward) moves backwards, “retreating” to another 1/3 of the cage*	Yes *(cat moved majority of body into a back third of the cage)* No *(cat did not move a majority of it’s body backward to another third of the cage)* At back *(cat was already at the back of the cage and could not step any farther back)*	Crack Cage Door Novel Object Interactive Toy
**Withdraw from rod** (at any time) *The cat's feet stepped away from the rod*	Yes *(cat stepped away)* No *(cat did not step away)* At back *(cat was already way at the back of the cage and could not step any further away and remained there throughout the assessment)*	Touch with Rod
**Yawn** (throughout assessment) *Cat opens the mouth widely with a prolonged, deep inhalation and sighing or heavy exhalation*	Yes No Unknown (Cat turned away) Unknown (Cat under/behind stuff)	Crack Cage Door Novel Object Interactive Toy Touch with Rod
